# Association between Dietary Patterns and Precocious Puberty in Children: A Population-Based Study

**DOI:** 10.1155/2018/4528704

**Published:** 2018-01-16

**Authors:** Chang Chen, Yao Chen, Yunting Zhang, Wanqi Sun, Yanrui Jiang, Yuanjin Song, Qi Zhu, Hao Mei, Xiumin Wang, Shijian Liu, Fan Jiang

**Affiliations:** ^1^Pediatric Translational Medicine Institute, Shanghai Children's Medical Center, Shanghai Jiao Tong University School of Medicine, Shanghai, China; ^2^School of Public Health, Shanghai Jiao Tong University School of Medicine, Shanghai, China; ^3^Department of Endocrine and Genetic Metabolic Diseases, Shanghai Children's Medical Center, Shanghai Jiao Tong University School of Medicine, Shanghai, China; ^4^Shanghai Key Laboratory of Children's Environmental Health, Xinhua Hospital, Shanghai Jiao Tong University School of Medicine, Shanghai, China; ^5^Child Health Advocacy Institute, Shanghai Children's Medical Center, Shanghai Jiao Tong University School of Medicine, Shanghai, China; ^6^Department of Developmental and Behavioral Pediatrics, Shanghai Children's Medical Center, Shanghai Jiao Tong University School of Medicine, Shanghai, China; ^7^Department of Data Science, University of Mississippi Medical Center, Jackson, MS, USA

## Abstract

**Objective:**

The aim of the present study was to investigate the association between dietary patterns and precocious puberty among Shanghai children.

**Methods:**

A cross-sectional study was conducted among Shanghai children by multistage stratified cluster random sampling in June 2014. Diet was assessed using a simplified food frequency questionnaire (FFQ). Height, weight, and Tanner stages of breast development, pubic hair growth, and testicular volume were carefully measured. Exploratory factor analysis was used to identify dietary patterns, and logistic regression analysis was used to assess the association between dietary patterns and precocious puberty.

**Results:**

Three distinct dietary patterns, “traditional diet,” “unhealthy diet,” and “protein diet,” were established. Neither the “traditional diet” pattern nor the “protein diet” pattern showed any association with precocious puberty, taking gender, BMI, and adjustment factors into consideration. The “unhealthy diet” pattern was significantly positively associated with precocious puberty in both boys (OR = 1.24, 95% CI = 1.02–1.51) and girls (OR = 1.31, 95% CI = 1.10–1.56). The relationship remained positive only for girls (OR = 1.25, 95% CI = 1.04–1.49) after adjustment for age and BMI but statistically nonsignificant after further adjustment for socioeconomic factors in both boys and girls.

**Conclusions:**

Dietary patterns were found to be related to precocious puberty among Shanghai children.

## 1. Introduction

Historical studies have demonstrated a decrease in the age at the onset of puberty [1–3]. Early onset of puberty may confer an intermediary factor on the life-course path to a number of adverse health consequences in later life including hormone-related cancers [4, 5], metabolic syndrome [6], and higher risks of all-cause mortality [7, 8]. Because of these important ramifications, modifiable factors that influence the timing of puberty are of endocrinological interest from a public health perspective.

Large numbers of epidemiological studies have suggested notable associations between dietary intake and pubertal timing besides energy imbalance. Children with the highest intakes of vegetable protein experienced pubertal onset up to 7 months later, and those with the highest intake of animal protein experienced it up to 7 months earlier [9]. A delay in menarcheal age was observed in relation to higher fiber intake in childhood [10]. Consumption of sugar-sweetened soft drinks is also positively associated with the risk of earlier puberty [11, 12]. This suggests that a childhood diet is a modifiable factor and that the timing of pubertal onset and environmental exposure may be amenable to intervention.

None of these dietary factors acts alone. Rather, a combination of nutritional influences partially directs the timing of puberty [13]. Accordingly, evaluating dietary patterns can be used as a semiquantitative method to explore the overall relationship between diet and disease. It may be suitable for large-scale epidemiological studies. The Eastern diet pattern is quite different from the Western diet pattern, and the influence of the Eastern diet pattern on precocious puberty is limited. Further insight into Eastern dietary factors might influence the onset of pubertal development. The current work was designed to explore the combined effects of dietary patterns on precocious puberty in 6- to 12-year-old schoolchildren in China.

## 2. Materials and Methods

### 2.1. Participants and Sampling

Participants were selected from the Shanghai Children's Health, Education, and Lifestyle Evaluation (SCHEDULE) study. Details of the sampling scheme and recruitment procedure are given as follows. A cross-sectional study was conducted in June 2014 using school-based, multistage, stratified cluster random sampling in a central urban area, inner suburb, and outer suburb, as classified in the 2005 Shanghai census report (http://www.stats.gov.cn/tjgb/rkpcgb/dfrkpcgb/t20060320_402311562.htm). A total of 26 general primary schools in 7 districts, Changning, Jing'an, Zhabei, Jinshan, Pudong, Jiading, and Chongming, were randomly sampled from school lists, and students in grades 1 to 5 were recruited. Children who were taking medication capable of causing precocious puberty were excluded from the study. Informed consent was obtained from the participant children and their parents, and the study was approved by the Institutional Review Boards of the Shanghai Children's Medical Center.

### 2.2. Dietary Assessment

An adaptation of the simplified food frequency questionnaire (FFQ), based on our precious study [14], was adopted to obtain the frequency information of food intake over the past 6 months. The FFQ was based on the most frequently consumed foods that were clinically considered possibly related to precocious puberty, including vegetables, fruit, red meat, white meat, aquatic and sea food, desserts and snacks, fried foods, neogala, protein powder, dairy product, and soft drinks with a scale covering 5 levels of food intake frequency. Trained teachers handed out the questionnaires to the recruited students, asking them to take the questionnaires home and have their parents fill it in. Then teachers collected the completed questionnaires and returned them to the investigators. Based on the distribution of frequency, each food item was recoded into 2 or 3 categories to analyze the impact of single food on precocious puberty.

### 2.3. Anthropometry

The Tanner staging method was used to assess children's sexual development, jointly evaluated by child health physicians and pediatric endocrinologic physicians. Breast development was determined using inspection combined with palpation, and testicular volume was assessed by palpation and a Prader testicular meter. Breast and pubic hair developments were graded from 1 to 5. The Tanner stage was delineated by Marshall and Tanner [15, 16]. Precocious puberty was defined as Tanner stage 2 for breast development (B_2_) or pubic hair development (PH_2_) under the age of 8 in girls and as PH_2_ or testicle development (T_2_) (testicular volume (TV) ≥ 4 ml) under the age of 9 in boys [17–19].

A stadiometer to the nearest 0.1 cm and a standardized digital scale to the nearest 0.1 kg were used to measure height and weight while the children were barefoot and wearing light clothes. Both height and weight were measured twice, and a third measurement was taken if the first 2 differed by as much as 1.0 cm or 1.0 kg. After the third measurement was taken, the closest 2 measurements were averaged. BMI was calculated as weight (kg) divided by the square of height (m^2^).

### 2.4. Parental and Socioeconomic Characteristics

Parental and socioeconomic characteristics were corrected using a self-reported questionnaire and categorized as follows: total family income (low: <50,000 Chinese yuan per year; middle: 50,000–150,000 Chinese yuan per year; and high: ≥150,000 Chinese yuan per year), parental education (low: middle school or below; middle: high school or technical school; and high: college degree or more), and area (urban districts: Changning, Jing'an, and Zhabei; suburban districts: Jinshan, Pudong, Jiading, and Chongming) [20].

### 2.5. Statistical Analysis

Data were duplicated entry and proofread using EpiData 3.1 (EpiData Association, Odense, Denmark) by 2 groups of researchers and analyzed using IBM SPSS 21.0 (IBM Corp., Armonk, NY, U.S.). Qualitative data here are expressed as frequency (%), and quantitative data as mean ± standard deviation (SD). The student *t*-test and chi-square test were performed to assess differences among subgroups.

Based on the results of Kaiser-Meyer-Olkin (KMO) and Bartlett's test, exploratory factor analysis was conducted to identify major dietary patterns. The principal components were identified according to the scree plot, and factor loading values ≥ 0.3 or ≤−0.3 were considered to make a significant contribution threshold to the pattern [21]. The factor scores of each pattern were calculated for each individual using principal component analysis with varimax rotation. The association between dietary patterns and precocious puberty was assessed using logistic regression analysis with the factor score, and odds ratios (OR) with 95% confidence intervals (CI) were calculated. Adjustments were further performed using the multivariate regression models—model I: unadjusted; model II: adjusted for age, gender, and BMI; and model III: further adjusted for socioeconomic variables.

## 3. Results

A total of 17,620 children were recruited, and 16,958 (96.24%) completed the physical examination. 15,937 (90.45%) children remained after data cleaning of the cases with missing key variables, including age, gender, weight, and height. On account of the age-specific definition of precocious puberty, it was only possible to determine whether 6254 children, including 4221 boys (67.49%) aged 6 to 9 (7.86 ± 0.65) years and 2033 girls (32.51%) aged 6 to 8 (7.44 ± 0.55) years had undergone precocious puberty or not. A total of 596 children (127 boys and 469 girls) were found to have undergone precocious puberty.

Descriptive information regarding socioeconomic and dietary factors is given in Tables 1 and 2. There were different distributions of socioeconomic and dietary factors among boys and girls. For example, girls took in more fruits and vegetables while boys ate more fried foods (*P* < 0.05), and Mather's education level was higher for girls than boys (*P* < 0.05). In this way, gender was found to be a critical confounder in the relationship between the precocious puberty and dietary pattern. The boys were taller (129.71 ± 6.59 cm) and heavier (28.60 ± 6.75 kg) than girls (125.78 ± 6.75 cm, 25.09 ± 5.79 kg; *P* < 0.05). BMI was significantly higher in boys (16.85 ± 2.90 kg/m^2^) than girls (15.72 ± 2.34 kg/m^2^) and in precocious children (17.63 ± 2.99 kg/m^2^) than normal puberty children (16.36 ± 2.73 kg/m^2^) (*P* < 0.001).

Kaiser-Meyer-Olkin measure of sampling adequacy was 0.69, and *P* value for Bartlett's test was less than 0.001, so exploratory factor analysis was considered suitable for the current study. The factor loading values accounted for 19.24%, 16.42%, and 11.64%, accounting for 47.31% of the total variation. Factor loading for dietary patterns is shown in Table 3. The three dietary patterns were identified as follows: the first dietary pattern (factor 1) was termed the “traditional diet”, in which vegetables, fruit, red meat, white meat, and aquatic and sea food showed a high positive loading. The second dietary pattern (factor 2) showed a high loading for dessert/snacks, soft drinks, and fried food and here was called the “unhealthy diet.” The third dietary pattern (factor 3) was called the “protein diet.” It showed the highest positive loading for neogala, protein powder, and dairy products.

The association between the dietary pattern and precocious puberty is given in Table 4. As for total children, none of the models showed any statistically significant differences. Though age and gender were covariant in the current model, detailed analysis of the multivariate logistical regression results was performed for all age groups. For boys, although the traditional diet pattern showed a protective association with precocious puberty among 6-year-olds, the range of 95% CI was relatively wide (OR = 0.23, 95% CI = 0.00–1.51). In the 8-year-old group, the unadjusted regression model showed that higher dietary factor scores for the unhealthy diet pattern were significantly closely associated with the higher risk of precocious puberty (OR = 1.24, 95% CI = 1.02–1.51). The protein diet pattern was not found to be statistically related to precocious puberty (*P* > 0.05). Among girls, there was no observable association between precocious puberty and either the traditional diet pattern or the protein diet pattern (*P* > 0.05), but the unhealthy diet pattern was found to be significantly closely associated with the higher risk of precocious puberty in the unadjusted regression model (OR = 1.31, 95% CI = 1.10–1.56). After adjusting for age and BMI, the unhealthy diet pattern was still positively associated with precocious puberty (OR = 1.25, 95% CI = 1.04–1.49). Nonetheless, after further adjustment for socioeconomic confounders, none of the 3 major dietary patterns showed any statistically significant association with precocious puberty.

## 4. Discussion

The present study was a cross-sectional study to explore the association between dietary and precocious puberty in 6- to 12-year-old children in Shanghai, China. Three major dietary patterns were identified using exploratory factor analysis: traditional diet, unhealthy diet, and protein diet. The unhealthy diet pattern was found to be significantly closely associated with precocious puberty in both girls and boys. The 3 dietary patterns contributed to 47.31% of the total variation. These findings are similar to those of a previous study that identified 4 components from food frequency data that explained 29.2% of the dietary variation [21].

The current findings hold that the traditional diet pattern, which is heavy on vegetables, fruits, red meat, white meat, and aquatic and sea food, was not associated with sexual development in either boys or girls after adjustment for confounders, because the only positive results in boys might be ascribed to the small sample size of the 6-year-old group. It was hypothesized that greater consumption of fruit and vegetables could be related to later pubertal onset among girls because of 2 phytoestrogens, lignin, and flavonol [13]. Some evidence indicated that a higher meat intake during childhood might be related to the earlier pubertal onset due to a protein-mediated enhancement of growth factor expression [2, 22]. Li et al. reported in a cross-sectional study of 422 Korean children that a “shellfish and processed meat” dietary pattern was found to be weakly positively associated with genital development in boys (OR = 1.65, 95% CI = 0.95–2.89) and significantly positively related to breast development in girls (OR = 1.88, 95% CI = 1.08–3.26) after adjustment for confounders [21]. Similarly, in a cohort study, Jansen et al. reported that girls who consumed red meat ≥ 2 times/day had a significantly earlier age at menarche than those who consumed red meat < 4 times/week [23]. In this way, the frequency of intake of red meat was shown to be inversely related to age at menarche (HR = 1.64, 95% CI = 1.11–2.41). Conversely, in a prospective cohort study of 109 healthy Caucasian children, Remer et al. deduced that independent adrenarchal androgen secretion, rather than animal protein intake, precipitates an earlier onset of breast, genital, and pubic hair development [24]. Notably, other nutrients present in animal foods, specific micronutrients and fatty for instance, might also play a role in the timing of puberty.

The unhealthy diet pattern, heavy in desserts and snacks, soft drinks, and fried food, was found to be significantly positively associated with precocious puberty in both boys and girls. The association remained positive and significant in girls after adjustment for age and BMI but not in boys. However, no significant association was observed after adjustment for socioeconomic factors, which indicates that the magnitude of a socioeconomic effect was likely to be nonnegligible. This diet was implicated in the timing of puberty, probably in any of the 3 ways: high fat intake, high sugar, and obesity due to high-calorie consumption. Consumption of junk food, such as fried foods, had been convincingly linked to obesity and rapid weight gain, a potential predictor of earlier age at menarche and other markers of puberty [22, 25]. However, energy intake from carbohydrates during childhood has not been found to show any association with markers of pubertal development in cohort studies, such as breast development and menarche [26–28]. In this way, an unhealthy diet, independent of obesity, might also lead to precocious puberty. Various studies have shown that fat intake has a potential influence on estrogen metabolism and might be related to the time of the onset of puberty [29]. Taken together, higher total fat intake and total polyunsaturated fatty acid intake were linked to earlier menarche [30, 31]. However, saturated fatty acids [32], monounsaturated fatty acids [33], and animal fat were found to be related to later menarche [10], but the mechanism underlying this relationship remains unclear and the pattern has been difficult to interpret [9]. Soft drinks were generally sugar- and artificially sweetened beverages capable of causing immediate increases in circulating insulin concentrations. Studies found more frequent soft drink consumption to be predictive of earlier menarche through metabolic changes in insulin-mediated pathway mechanisms and upregulation of hormones, in addition to increased BMI [11, 12, 34].

The protein diet pattern showed the high positive loading for neogala, protein powder, and dairy products and tended not to be associated with precocious puberty in the current study. The effects might vary according to protein sources [22]. The main ingredient in protein power was vegetable protein, and observational studies have found vegetable protein intake in childhood to be positively related to an earlier onset of puberty. Gunther et al. that reported children aged 3-4 years with higher vegetable protein intake experienced menarche or voice break approximately 0.5 years later [35]. Similarly, Berkey et al. detected an association of a 1 SD higher vegetable protein intake (approximately 3 g/day) with menarche 0.87 years later in girls aged 3–5 years [36]. Current evidence suggests that dietary protein intake from milk and dairy products might be implicated in an earlier onset of puberty by stimulating the secretion of IGF-1 [9, 35, 37–39]. Other studies have reported null findings from milk supplementation [31, 38, 40]. On account of the complex nutrition and multiple hormonally active substances of dairy product, the overall effect on the timing of puberty remains unknown.

The effect of dietary intake on puberty development is still uncertain and inconsistent. One possible explanation for this discrepancy is incongruent timing and method of dietary assessments. The accuracy of qualitative and quantitative measurements can differ, and diet around the time of puberty onset may be less relevant than prepuberty [23]. Multiple components of food including natural components and additives and underlying interaction may effect influence on puberty timing.

In light of these strengths, earlier studies seldom discussed the dietary pattern, focusing mainly on the single effects of foods or nutrients on the timing of puberty. However, foods are consumed in combination and contain more than 1 nutrient. For this reason, the overall eating pattern is what affects sexual maturation. In the current study, this is especially true for boys. Moreover, our study is generalizable to children in Shanghai because of strict sampling methods and strong representation of all socioeconomic levels.

The current investigation also has some limitations. First, based on the inability to estimate the time order of diet and disease, no causal relationship between dietary pattern and precocious puberty could be proven because of the cross-sectional design. The FFQ used in our study measured food consumption over the previous 6 months. In real life in China, school-age children have relatively stable habits. Their dietary patterns might change less than those of children in other countries because of the unchanged family condition and fixed dependents. The dietary habits predicted by the FFQ might be even longer than half of a year. Large prospective cohort studies are needed to confirm the causal association. Second, compared with 24-hour dietary recall or diet weight measurement, the FFQ assessed by self-reporting inevitably led to recall bias, which may have affected the accuracy of the current study. We plan to use more accurate methods of dietary evaluation in our further work. Third, the diagnosis of precocious puberty, based on inspection and palpation by pediatric endocrinology physicians and pediatric care physicians, may also have created false-positive results. In this way, further studies involving diagnostic imaging such as B-ultrasonic scans are needed to build on these findings.

In summary, the current study focused on the role of the 3 dietary patterns in precocious puberty. The unhealthy diet pattern was found to be significantly positively associated with precocious puberty in both boys and girls, but these results were found to be statistically insignificant after adjustment for age and BMI for boys and further adjustment for socioeconomic factors for girls. Neither the traditional dietary pattern nor the protein dietary pattern showed any association with precocious puberty after gender and adjustment factors were taken into consideration. These findings may provide further support for public health efforts to guide a balanced diet and reduce junk food consumption and offer insight into the prevention and control of precocious puberty.

## Figures and Tables

**Table 1:** Characteristics of the study population.

Variables	Boys (*N*) (%)	Girls (*N*) (%)	*P* value
Precocious puberty	127 (3.01%)	469 (23.07%)	<0.001
Socioeconomic factor			
Father's education level			0.001
Low	1251 (30.51%)	519 (26.13%)	
Middle	1185 (41.59%)	588 (29.61%)	
High	1664 (58.41%)	879 (44.26%)	
Mother's education level			0.010
Low	1426 (35.14%)	611 (31.22%)	
Middle	1068 (26.32%)	538 (27.49%)	
High	1564 (38.54%)	808 (41.29%)	
Total family income			0.587
Low	806 (28.11%)	382 (26.75%)	
Middle	1340 (46.74%)	672 (47.06%)	
High	721 (25.15%)	374 (26.19%)	
District			0.461
Suburban	3491 (82.71%)	1666 (81.95%)	
Urban	730 (17.29%)	367 (18.05%)	

*Notes*. *P* value for chi-square test.

**Table 2:** Dietary factors associated with precocious puberty as assessed by univariate analysis.

Variables	Boys (*N*) (%)	Girls (*N*) (%)	*P* value
Vegetable			<0.001
≤3 times/week	718 (17.55%)	265 (13.34%)	
4–6 times/week	513 (12.54%)	271 (13.65%)	
≥1 times/day	2860 (69.91%)	1450 (73.01%)	
Fruit			0.010
≤3 times/week	921 (22.51%)	380 (19.18%)	
4–6 times/week	796 (19.46%)	416 (21.00%)	
≥1 times/day	2374 (58.03%)	1185 (59.82%)	
Red meat			0.178
≤3 times/week	1408 (34.59%)	729 (36.95%)	
4–6 times/week	795 (19.53%)	381 (19.31%)	
≥1 times/day	1867 (45.87%)	863 (43.74%)	
White meat			0.224
≤1 time/week	1025 (25.21%)	538 (27.27%)	
1–3 times/week	2087 (51.33%)	990 (50.18%)	
≥4 times/week	954 (23.46%)	445 (22.55%)	
Aquatic and sea food			<0.001
≤1 time/week	965 (23.75%)	379 (19.17%)	
1–3 times/week	2207 (54.31%)	1130 (57.16%)	
≥4 times/week	892 (21.95%)	468 (23.67%)	
Dessert/snacks			0.002
≤1 time/week	864 (21.22%)	352 (17.78%)	
1–3 times/week	1732 (42.54%)	837 (42.27%)	
≥4 times/week	1475 (36.23%)	791 (39.95%)	
Fried food			0.006
≤1 time/month	1568 (38.56%)	831 (42.01%)	
2-3 times/month	1646 (40.48%)	792 (40.04%)	
≥1 time/week	852 (20.95%)	355 (17.95%)	
Neogala			0.920
No	3529 (87.35%)	1720 (87.44%)	
Yes	511 (12.65%)	247 (12.56%)	
Protein powder			0.151
No	3487 (86.44%)	1730 (87.77%)	
Yes	547 (13.56%)	241 (12.23%)	
Dairy product			<0.001
≤100 ml/day	872 (21.69%)	505 (25.88%)	
100–300 ml/day	2153 (53.56%)	1115 (57.15%)	
≥300 ml/day	995 (24.75%)	331 (16.97%)	
Soft drinks			<0.001
≤3 times/month	1741 (42.93%)	941 (47.67%)	
1–3 times/week	1590 (39.21%)	742 (37.59%)	
≥3 times/week	724 (17.85%)	291 (14.74%)	

*Notes*. *P* value for chi-square test.

**Table 3:** Factor loading matrix for dietary patterns by exploratory factor analysis.

Variable	Traditional diet pattern	Unhealthy diet pattern	Protein diet pattern
Vegetable	**0.631**	−0.249	−0.06
Fruit	**0.675**	−0.113	0.033
Red meat	**0.637**	0.204	−0.138
White meat	**0.585**	**0.366**	0.086
Aquatic and sea food	**0.634**	0.149	0.109
Dessert/snacks	0.102	**0.713**	−0.007
Fried food	0.010	**0.664**	−0.005
Neogala	0.024	0.078	**0.751**
Protein powder	−0.080	0.052	**0.747**
Dairy product	**0.307**	−0.049	**0.316**
Soft drinks	−0.034	**0.758**	0.126
Eigenvalue	2.117	1.807	1.280
Percentage of variability	19.244	16.424	11.640

*Notes*. Absolute factor loading values > 0.30 were labeled in bold.

**Table 4:** Multivariate logistical regression for dietary patterns and precocious puberty.

	Traditional diet pattern		Unhealthy diet pattern		Protein diet pattern	
	OR (95% CI)	*P* value	OR (95% CI)	*P* value	OR (95% CI)	*P* value
Total						
Model I	1.01 (0.92–1.10)	0.882	1.06 (0.97–1.16)	0.171	0.92 (0.84–1.01)	0.092
Model II	1.01 (0.92–1.10)	0.903	1.04 (0.95–1.14)	0.383	0.92 (0.83–1.01)	0.076
Model III	0.93 (0.83–1.04)	0.193	0.97 (0.86–1.08)	0.524	0.96 (0.85–1.08)	0.451
Boys						
*6 years old group*						
Model I	0.48 (0.13–1.73)	0.476	1.38 (0.44–4.33)	0.579	1.13 (0.36–3.52)	0.831
Model II	0.19 (0.02–1.45)	0.108	1.35 (0.42–4.35)	0.612	0.93 (0.20–4.32)	0.923
**Model III**	**0.23 (0.00–70.57)**	**0.001**	20.28 (0.40–1027.11)	0.133	1.23 (0.16–9.45)	0.843
*7 years old group*						
Model I	1.03 (0.65–1.62)	0.915	1.43 (0.93–2.18)	0.104	0.98 (0.62–1.53)	0.915
Model II	1.03 (0.65–1.63)	0.898	1.42 (0.92–2.17)	0.113	0.98 (0.62–1.54)	0.929
Model III	0.91 (0.50–1.67)	0.769	1.04 (0.82–1.32)	0.740	1.04 (0.60–1.80)	0.885
*8 years old group*						
Model I	0.87 (0.71–1.07)	0.190	**1.24 (1.02–1.51)**	**0.033**	1.10 (0.91–1.32)	0.331
Model II	0.88 (0.71–1.08)	0.214	1.21 (0.99–1.48)	0.057	1.10 (0.91–1.32)	0.337
Model III	0.89 (0.67–1.19)	0.431	1.04 (0.80–1.36)	0.762	1.11 (0.86–1.44)	0.405
Girls						
*6 years old group*						
Model I	1.15 (0.88–1.51)	0.318	1.05 (0.80–1.38)	0.742	0.82 (0.57–1.16)	0.253
Model II	0.95 (0.71–1.28)	0.753	1.02 (0.76–1.37)	0.898	0.88 (0.61–1.28)	0.504
Model III	0.88 (0.59–1.33)	0.542	0.98 (0.66–1.46)	0.910	1.17 (0.72–1.89)	0.532
*7 years old group*						
Model I	0.88 (0.73–1.06)	0.189	**1.31 (1.10–1.56)**	**0.003**	0.91 (0.80–1.04)	0.161
Model II	0.89 (0.74–1.07)	0.210	**1.25 (1.04–1.49)**	**0.015**	0.89 (0.77–1.02)	0.098
Model III	0.87 (0.67–1.12)	0.264	1.04 (0.82–1.32)	0.740	0.97 (0.82–1.16)	0.739

*Notes*. *P* < 0.05 were labeled in bold. Model I: unadjusted. Model II: adjustment for age and BMI. Model III: adjustment for father's education level, mother's education level, total family income, and district based on model II.
